# The health effects of gendered and devalued work: health outcomes of incarcerated women engaging in sex work and care/service work

**DOI:** 10.1186/s40352-020-00124-4

**Published:** 2020-11-18

**Authors:** Sage J. Kim, Caryn Peterson

**Affiliations:** 1 Division of Health Policy & Administration, University of Illinois at Chicago, School of Public Health, 1603 W. Taylor St. #781, Chicago, IL USA; 2 Division of Epidemiology & Biostatistics, University of Illinois at Chicago, School of Public Health, Chicago, IL USA

**Keywords:** Incarcerated women, Sex work, Care work, Health of women in jail

## Abstract

**Background:**

Women with a history of incarceration are often engaged in highly gendered work, either sex work or low-wage care/service work jobs. While employment is an important element of reentry plans, low-wage jobs may not necessarily help women leave illicit activities, including commercial sex work. Incarcerated women often move between care/service work and sex work to supplement income, putting them at greater risk for negative health outcomes.

**Results:**

Using survey data from 400 women detained in a large urban jail, we examined how incarcerated women’s experience with sex work and low-wage care/service work affects four health-related outcomes: overall health concerns, clinical depression, regular drug use, and self-esteem. Of the survey participants, 24% engaged exclusively in sex work and 34% in care/service work. However, 41% of women held both sex work and care/service work jobs, prior to incarceration. Compared to women engaged in care/service work, a greater proportion of women engaged in sex work reported overall health concerns, clinical depression, and regular drug use. On the other hand, women in care/service work jobs exclusively reported lower levels of self-esteem than women engaging in sex work.

**Conclusions:**

Many reentry programs emphasize the importance of employment for former inmates, and yet, job options for women detained in jail are often limited to low-wage care/service jobs, which do not necessarily provide adequate security to lift women’s economic burdens. Consequently, many women with a history of incarceration may supplement their income with sex work to meet their basic economic needs. However, both of these highly gendered and devalued jobs may negatively affect health and wellbeing of women.

## Introduction

Currently, 6.7 million Americans are either incarcerated in jails and prisons, or under community supervision, representing approximately 2.8% of the U.S. population (Jones, 2018). Although women account for a relatively small proportion of the incarcerated population, the number of women in correctional facilities has been increasing at a rate 50% higher than men since 1980 (The Sentencing Project, 2016). More than 1.3 million women are currently held in the criminal justice system. In 2017, women comprised 7.6% of the prison population and 15.1% of the jail population (Jones, 2018). In Cook County, Illinois, where Chicago is located, approximately 6.6% of 5600 daily county jail detainees are women (Cook County Department of Corrections, 2019; Cook County State’s Attorney Office, 2019). Incarceration significantly weakens one’s employment opportunities (Alexander, 2010; Curtis, 2011; Morenoff & Harding, 2014; Uggen & Manza, 2002; Western & Pettit, 2005; Western & Wildeman, 2009). The disproportionate increase in incarceration rates in minority communities since the 1980s has meant that a large number of low-skilled minority workers are trapped in low-wage and/or temporary jobs, or excluded entirely from the labor force (Nagin & Waldfogel, 1995; Sampson & Laub, 1993; Western, 2007; Western & Beckett, 1999).

Although the negative effects of incarceration on employment have been widely documented, the employment conditions of incarcerated women and the related health effects have been understudied (Cobbina, 2009; Cox, 2012; Nuytiens & Christiaens, 2016). Women with a history of incarceration struggle to find jobs that provide a living wage (Bergseth, Richardson Jens, Bergerson-Vigesaa, & McDonal, 2011; Decker, Spohn, Ortiz, & Hedberg, 2014; Wright, Van Voorhis, Salisbury, & Bauman, 2012), and many of these women resort to exchanging sex for money (McClanahan, McGlelland, Abram, & Teplin, 1999; Riley, Gandhi, Hare, Cohen, & Hwang, 2007). Certainly, incarceration affects future employment outcomes, but it is also true that multiple social and economic factors that lead women to come in contact with the criminal justice system contribute to limited employment opportunities and poverty (Bergseth et al., 2011; Decker et al., 2014; Kim, 2003; McClanahan et al., 1999; Riley et al., 2007; Wright et al., 2012).

The current low-wage care work economy has contributed to further deterioration of economic conditions for the poor (Iceland, 2013; Rosaldo, Tilly, & Evans, 2012; Smith & Halpin, 2011). Individuals with a history of incarceration are additionally burdened by limited economic opportunities and are often locked in low-wage precarious jobs, while poor economic conditions increase the likelihood of incarceration/recidivism (Centers for Disease Control and Prevention, 2016; Chen, McFarland, & Raymond, 2011; Cohan et al., 2005; Salisbury & Van Voorhis, 2009). Job training and work-related interventions are frequently offered as components of reentry programs for women returning from corrections (Latessa, 2012; Solomon, Deadel Johnson, Travis, & McBride, 2004). However, job options for low-skilled women with a history of incarceration are limited (Western, 2002), and the lack of a living wage may result in these women seeking supplemental income through illicit work (Nightingale & Wandner, 2011). Sex work and low-wage care/service work are generally conceptualized as distinct categories of employment with little overlap. However, we argue that incarcerated women move between the two types of jobs more fluidly than previously understood and hypothesize that these jobs are associated with poorer health outcomes. The objective of this study was to examine how incarcerated women’s experience with sex work and low-wage care/service work affects health and wellbeing, as measured by overall health concerns, clinical depression, regular drug use, and self-esteem.

### Gendered sex work

The Federal Bureau of Investigation (FBI) Uniform Crime Report (UCR) shows that about 1% of more than 2.2 million female arrests nationwide were for prostitution in 2015 (Federal Bureau of Investigation, 2000). In Cook County, Illinois, which includes Chicago, just over 7% of female detainees in Cook County Jail were incarcerated for prostitution (Escobar & Olson, 2012). However, these figures vastly underrepresent the number of women in jail who exchange sex for money or drugs (Harris et al., 2003; Kim, Johnson, Goswami, & Puisis, 2011). For example, Binswanger and others documented that 56% of former female inmates in their study had engaged in sex exchange (Binswanger, Mueller, Beaty, Min, & Corsi, 2014). Similarly, Kim and colleagues found that close to 45% of female detainees in Cook County Jail reported having exchanged sex for money or drugs (Kim et al., 2011).

To understand women’s path into sex work, scholars have explored two different perspectives: the “oppression” paradigm and the “empowerment” paradigm (Weitzer, 2010). The oppression paradigm highlights the effects of negative childhood experiences, including physical and sexual abuse, family instability, poverty, homelessness, and drug use on later commercial sex work. Conversely, the empowerment perspective recognizes that more immediate circumstances, such as current economic needs, human capital, and limited employment opportunities contribute to women’s decisions to engage in commercial sex work. The empowerment paradigm further argues that lack of education, work experience, or skills contribute to a greater willingness to engage in commercial sex work to make up for the lack of occupational opportunities.

The forces behind these two opposing perspectives may be highly intertwined, shaping women’s trajectory into sex work (Agustín, 2006; McCarthy, Benoit, & Jansson, 2014; Weitzer, 2010). A myriad of factors contribute to women’s limited job options, and the decision to enter into sex work range from survival needs to a desire for financial independence (Weitzer, 2009). For example, McCarthy and colleagues found that both negative experiences from early life and immediate life circumstances increase the likelihood of engaging in sex work as opposed to low-wage care/service work (McCarthy et al., 2014).

Other scholars suggest that women might choose to enter into commercial sex work as a way of escaping the oppressive conditions that they experience in low-wage domestic work or unemployment (Bernstein, 2004; Murphy & Venkatesh, 2006; Raphael & Shapiro, 2002). Sex work becomes a way to supplement their income, buffering them from the conditions that restrict their employment options in the formal economy (Bernstein, 2004; Raphael & Shapiro, 2002). Rosen and Venkatesh (2008) argue that commercial sex work is a part of a set of resource exchange strategies within a continuum of low-wage jobs and the underground economy. Levitt and Venkatesh estimate that street sex workers in Chicago earn about $27 per hour, which is substantially higher than the average earnings of low-wage female care workers with earnings at $11 per hour (Bureau of Labor Statistics, 2017; Edlund & Korn, 2002; Levitt & Venkatesh, 2008).

### Gendered care/service work

The U.S. has experienced a period of “great divergence” (Noah, 2012) since the 1990s, where the wage gap between “good” and “bad” jobs has increased dramatically (Kalleberg, 2011). Many of the fastest growing low-wage jobs are characterized as care work which involves labor to help others enhance their physical, emotional, and developmental capabilities and includes child care, elderly care, home health aides, and social/welfare jobs (Bureau of Labor Statistics, 2017). Some researchers include related service work in their definition of paid care work, including domestic maids or food service workers, or jobs that address body and beauty, such as hairdressers and cosmetologists (England, Budig, & Folbre, n., 2002; Folbre, 2008).

Care work is, for the most part, a highly gendered form of work. Because many caring tasks that form the basis of care work were traditionally performed by female family members in the home for free, current paid care work is associated with woman’s nurturing characteristics, rather than a set of skills (Dwyer, 2013; England, 2005; England et al., 2002). As a result, care work is often undervalued and results in low-wages (Kilbourne, England, Farkas, Beron, & Weir, 1994; Levanon, England, & Allison, 2005; Meyer, 2000; Romero & Pérez, 2016; Steinberg, 1990). Low-wage care work has become unregulated and unprotected (England et al., 2002; Moller & Rubin, 2008; Rosaldo et al., 2012). The often precarious conditions of care workers create the need for secondary sources of income through informal and/or underground economic activities (Bales, 1984; Edin & Lein, 1997), blurring the line between the low-wage labor market and the underground economy (Nightingale & Wandner, 2011; Venkatesh, 2006).

### Nexus between paid care/service work and commercial sex work

Scholars have used life course approaches to examine women’s distinct life events and experiences which lead to either low-wage care/service work or commercial sex work (McCarthy et al., 2014). However, many incarcerated women engage in the formal and informal economy simultaneously (Gunter, 2017). For example, the Urban Justice Center documents that former inmates had held legal jobs, such as civil service, construction work, babysitting, cleaning, and food service (The Urban Justice Center, 2005). At the same time, more than 67% of the former inmates in the study were not making enough money to survive which contributed to their subsequent involvement in sex work.

Women may not necessarily treat formal and informal work as mutually exclusive categories (Fagan & Freeman, 1999). Instead, they navigate both domains to balance resources and risks associated with each. For instance, higher income from illicit work may protect against low-wage work in the formal labor market, and the relative stability of the formal economy can provide a buffer against the physical and mental health risks of the informal economy, particularly illicit work. As scholars suggest, illicit activities may be explained as economic “rational” decisions to maximize one’s benefits (Sullivan, 1973; Sykes & Geller, 2016). In this way, illicit work, such as drug dealing or commercial sex work, may supplement, rather than replace jobs in the formal economy (Goffman, 2014; Levitt & Venkatesh, 2000; Murphy & Venkatesh, 2006). Although the literature demonstrates that incarcerated women utilize formal and informal work to maximize economic gains, the impact of such work arrangements on their physical and mental health has not been explored.

### Work and the health conditions of incarcerated women

Studies have shown that sex work and low-wage work separately have a myriad of negative health effects. Mental and physical health effects of sex work have also been well documented (Cohan et al., 2006; Puri, Shannon, Nguyen, & Goldenberg, 2017). In particular, the increased risk of sexually transmitted infections (STIs) among women engaging in sex work has been extensively examined (Leichliter, Dittus, Copen, & Aral, 2019; Park et al., 2019; Paz-Bailey, Noble, Salo, & Tregear, 2016). In addition, smoking and other substance use are known to be prevalent in women in sex work jobs (Cohan et al., 2005).

Relatively little research is available concerning the health of low-wage care workers, but current studies indicate that low-wage workers disproportionately suffer from work place injuries (Steege, Baron, Marsh, Chaumont Menendez, & Myers, 2014), hazard exposure, stress, job insecurity (Burgard & Lin, 2013; Kinder, 2020; Landsbergis, Grzywacz, & LaMontagne, 2014), harassment, and exploitation (Okechukwu, Souza, Davis, & de Castro, 2014).

As we argued previously, sex work and low-wage care work may not necessarily be mutually exclusive job prospects for incarcerated women. And yet, the health effects of women moving between two highly gendered and devalued job, low-wage care/service work and sex work, have not been explored. To address this gap, we examined differences in four health-related outcomes between incarcerated women engaged in sex work and low-wage care/service work in a large urban jail. We then discuss the theoretical and policy implications of women’s incarceration, gendered work, and health.

## Methods

### Setting

We conducted 400 in-person surveys with women incarcerated in the Cook County Jail (CCJ), located in Chicago, Illinois, which is one of the largest single-facility jails in the United States. To note, incarceration is the state of being confined, more specifically, confinement in a jail or prison (Bureau of Justice Statistics, 2018). A varying type and degree of institutions exist to incarcerate individuals convicted of crime. This confinement occurs before or after a criminal conviction. Unlike inmates in prisons, the vast majority of jail inmates are pre-trial detainees. Consequently, study participants were detained in CCJ for a relatively short time, on average 54 days (Chicago Appleseed, 2013). Only about 18% of those incarcerated in jail would go on to be sentenced to prison (Olson & Huddle, 2013; Olson & Taheri, 2012), and charges would be dropped in 15% of the cases. The remainder of those incarcerated in jail would be either sentenced to probation, considered time served or charge expired. The majority of CCJ detainees return to their communities after a short stay in jail, which provided us with the opportunity to examine current work conditions of women prior to their index incarceration. Survey data were collected as part of a larger study examining incarcerated women’s substance use, sexual risk, and other life experiences (DA 024012).

In-person surveys took place in the women’s divisions of CCJ between 2010 and 2014. Eligible participants had to be 20 years or older and able to provide consent. Two interviewers who were healthcare workers at CCJ and the principal investigator (PI) conducted surveys. The research team underwent University of Illinois’ Institutional Review Board (IRB) human subjects training. Interviewers recruited potential survey participants using screener questions. Women were excluded from the study if they had mental health issues severe enough to interfere with the ability to engage in an interview, as determined at the screening phase. Those who met the inclusion criteria and agreed to participate in the study were invited to a thorough review of informed consent. Considering the setting and the population, the research team ensured participants that their decision to participate or not would not affect their jail stay or treatment throughout all stages of the study (i.e., from screening to informed consent to the survey implementation). Participants were also reminded that they may choose not to answer any or all questions at any time. Throughout the study, the PI met with the research team regularly to discuss any concerns, as well as progress and interim findings. Interviews lasted approximately 1 h. Of the 400 women surveyed, 298 (74.5%) reported working prior to incarceration and 277 (final analytical sample) had worked in either care/service work or sex work.

### Variables

Job type was categorized into three groups: regular sex work, care/service work, and both regular sex work and care/service work. Women who described their involvement in sex exchange “routinely” or reported sex work as their occupation were classified as engaging in regular sex work. Care/service work included childcare, sick or elderly care, general caregiving, and medical assistant work, cleaning, housekeeping, waitressing, cooking, and hair/beauty service. Women who engaged in regular sex work as well as care/service work were classified as both.

Health and wellbeing outcomes included three dichotomous variables: having health concerns, clinical depression, and using drugs regularly; and one continuous variable: self-esteem. Women reported having mental and physical health problems and/or taking any medications for health problems were identified as having health concerns. Depression was measured using the Center for Epidemiologic Studies Depression Scale (CESD) which ranges from 0 to 60, and women scoring greater than 16 are classified as clinically depressed (American Psychological Association, 2017). Women reporting regularly using marijuana, cocaine, hallucinogens, heroin, or other illicit drugs were classified as regular drug users. The Rosenberg Self-Esteem Scale was used to measure self-esteem, which is a 10-item questionnaire with ranges between 10 and 40. Higher scores indicate higher self-esteem.

Sociodemographic characteristics included age, race/ethnicity (i.e., Black, Hispanic, and other), and education (i.e., less than high school education and high school education and above). Social support was measured using the Medical Outcomes Study (MOS) social support survey, which is constructed with 19 items, ranging from 0 to 100, with higher scores indicating greater levels of social support (Hays, Sherbourne, & Mazel, 1994; Sherbourne & Stewart, 1991). Adverse childhood events (ACEs) included reports of childhood physical or sexual abuse. In addition, incarceration status, which was a dichotomous variable (first time vs. more than one incarceration).

### Analysis

Differences in the distribution of demographic characteristics by job type were tested using Chi-square and t-test statistics for categorical and continuous variables, respectively. Multivariable logistic regression was used to examine the relationship between job type and the three dichotomous outcome measures: health concerns, clinical depression, and regular drug use. Multivariable linear regression was used to examine the relationship between job type and self-esteem. Multivariate models were adjusted for age, race/ethnicity, education, social support, ACEs, and first-time incarceration. Statistical analysis was performed using Stata® 15 (Stata Corporation LP, College Station, TX, USA).

## Results

### Sample characteristics

The majority (68.6%) of the 400 women participating in the original study were black, reflecting the racial/ethnic distribution of women in CCJ. Among the survey respondents, 298 women worked prior to incarceration and 277 reported engaging in regular sex work, or care/service work, or both sex and care/service work. We excluded 21 women who had other jobs, such as factory work, administrative jobs for the purpose of this analysis. The mean age of the women in our final sample (*N* = 277) was 37.7 (SD = 11.1) years. Just over 22.3% of women were incarcerated for the first time, with an average of 8.7 (SD = 9.0) incarcerations.

### Job type

The women who had worked prior to current incarceration (*N* = 298) reported 426 jobs, averaging 1.4 jobs per woman. Nearly 50% of the 426 reported jobs were illicit jobs (*n* = 25) or sex work (n-181). Over 17% of jobs were paid care work, including childcare, medical assistant, sick and elderly care, and house cleaning. In addition, 22% of jobs were service work including, hair/beauty care, waitress, restaurant cook or dishwasher, bartending, cashier, or sales. Just over 11% of jobs were administrative or labor including, factory, warehouse, administrative assistant, clerk/secretary, and construction. By far, paid care and service work, totaling 40% of jobs reported, dominated the type of jobs participants held before incarceration (Table 1).

**Table 1:** Types of jobs that women had prior to incarceration

Occupation	N (%)
Regular sex work^a^	181 (42.5)
Other illicit work^b^	25 (5.9)
Care work^c^	76 (17.8)
Service work^d^	95 (22.3)
Administrative & labor work^e^	49 (11.5)
Total reported jobs	426 (100)

Note: Job type

^a^Includes sex work occupation, regular sex exchange, prostitution, escort, or street walk

^b^Includes drug dealing, stealing, hustling, or panhandling

^c^ Includes childcare, sick or elderly care, medical assistant, or house cleaning

^d^Includes hair/beauty care, waitress, restaurant cook or dishwasher, bartending, cashier, or sales

^e^Includes factory, warehouse, administrative assistant, clerk/secretary, and construction work

Table 2 compares the characteristics of women who worked only in regular sex work, only in care/service work, and both sex and care/service work (*N* = 277). Women engaged in care/service work only were more likely to have less than high school education (37.0%) compared with women in sex work only (61.5%) or both (64.6%). In addition, women engaged solely in care/service work (60.9%) were less likely to have ACEs compared with women engaged in exclusively sex work (77.8%) or both sex work and care/service work (80.9%). The mean number of incarcerations was higher for women engaged exclusively in sex work (12.6), compared with care work only (5.8) and both work (8.9). There were no statistically significant differences in job type by age or race/ethnicity, or receipt of social support.

**Table 2:** Comparison of the distribution of characteristics by work engagement type (*N* = 277)

	Sex work only (***n*** = 117)	Care/Service work only (***n*** = 92)	Engaged in both (***n*** = 68)	***p*** value
Age^a^	38.4	37.7	40.5	n.s.
Race/Ethnicity				
Black	71.8	66.3	70.6	
Hispanic	6.8	9.8	5.9	n.s.
Other	21.4	23.9	23.5	
< High School Degree	61.5	37.0	64.6	<.01
Adverse childhood events	77.8	60.9	80.9	<.01
Social support^a^	63.5	70.2	65.9	n.s.
Incarceration				
Mean^a^	12.6	5.8	8.9	<.01
First time	8.5	32.6	16.2	
2–5	10.3	25.0	22.1	<.01
6–12	39.3	29.3	35.3	
> =13	41.9	13.0	26.5	
Health and wellbeing measures				
Health concerns	76.7	62.6	88.2	<.01
Clinical depression	81.0	64.8	82.1	<.01
egular drug use	81.0	42.4	72.7	<.01
Self-esteem^a^	28.0	26.6	27.6	<.01

^a^Mean comparison, otherwise proportional comparison

### Health outcomes by job type

**Fig. 1:**
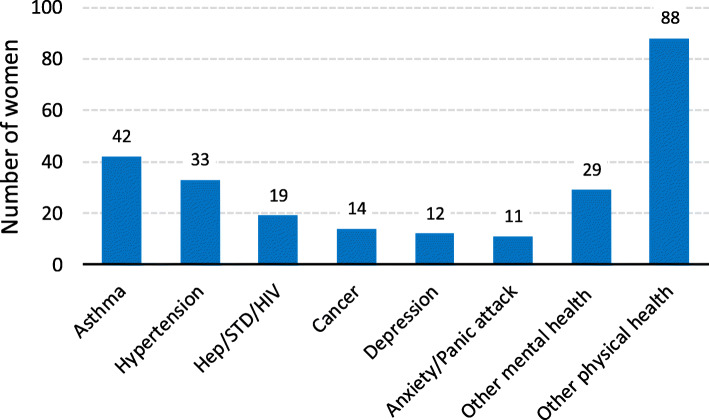
Mental and physical health concerns reported by incarcerated women (*N* = 248)

There were a total of 248 mental and physical health problems reported: 196 physical health and 52 mental health issues (Fig. 1). The most frequently reported health problems were asthma (*n* = 42) and hypertension (*n* = 33). Other physical health problems included: infectious diseases, cancer, diabetes, and injuries. Reported mental health problems included: depression (*n* = 12), anxiety or panic attack (*n* = 11), and other mental health issues (*n* = 29) including: bipolar disorder, and post-traumatic stress disorder (PTSD).

Table 2 summarizes the comparison among women by job type. A greater proportion of women engaged in both sex work and care/service work reported having health problems or currently taking medications (88.2%), than women in sex work only (76.7%) and care/service work only (62.6%). Women engaged in care/service work only were less likely to have clinical depression (64.8%) compared with women in sex work only (81.0%) and women working in both job types (82.1%). Similarly, women who worked exclusively in care/service work were less likely to be regular drug users (42.4%) than women engaging in sex work only (81.0%) or both (72.7%). On the other hand, women in care/service work had a lower level of self-esteem (mean = 26.6) compared with women in sex work only (mean = 28.0) and women engaged in both care/service and sex work (mean = 27.6).

Table 3 summarizes the results of regression models explaining the four health and wellbeing outcomes. After adjusting for age, race/ethnicity, education, social support, ACE, and first-time incarceration, women engaged in care/service work only were less likely to have health concerns, compared with women engaged in sex work only (*p* < 0.5). However, women engaged in both sex and care/service work were more likely than those in engaged in sex work only to have health concerns (*p* < .05). Women engaged exclusively in care/service work were less likely to have clinical depression (*p* < .05) or to be regular drug users (*p* < .01) than women engaging in sex work only. On the other hand, women engaged in care/service work only had lower levels of self-esteem compared to women engaged in sex work only (*p* < .05).

**Table 3:** Multivariate regression results explaining health and wellbeing outcomes

Variable	Health concerns	Clinical depression	Drug use	Self-esteem
	Odds Ratio			Coefficient
Age at interview	1.03	1.01	1.04*	1.63
Race/ethnicity				
All other (reference)	–	–	–	–
Black	0.74	1.17	0.39**	− 0.34
Social support	1.00	0.97**	0.99	−0.64
Less than HS education	0.50*	0.97	0.75	− 0.17
First time incarceration	1.76	2.66*	1.05	0.21
Adverse childhood events	0.85	1.43	0.71	−0.61
Work type				
Sex work only (reference)	–	–	–	–
Care/service only	0.73*	0.45*	0.13**	− 2.48*
Both	2.98*	1.22	0.51	− 0.71

*p < .05

**p < .01

## Discussion

This study examined incarcerated women’s engagement in sex work and low-wage care/service work and the impact of this work on their health and wellbeing. First, we found that more than 40% of women who reported working prior to incarceration engaged in both sex work and care/service work. This suggests that these two job categories are not mutually exclusive among incarcerated women. Early conceptualizations of domestic work among female European immigrants considered paid housework to be a “bridging occupation” (Romero & Pérez, 2016) that would allow women to transition into higher-paying, middle-class jobs. However, for women with a history of incarceration the transition from low-wage care work to better paying jobs has proven to be difficult (Brewer, 1999; Collins, 2000). The limited job options for women with a history of incarceration (Glenn, 2010; Romero & Pérez, 2016) force them into highly gendered and devalued work that cannot lift them out of poverty (Rabuy & Kopf, 2015; Sentencing Project, 2012).

Our study also demonstrated that incarcerated women have multiple health problems, and that overall health concerns were more prevalent among women engaging in sex work, compared to those engaged in care/service work. Women in sex work are disproportionately exposed to violence, sexually transmitted diseases, drug abuse, mental health problems, and exploitation (Anklesaria & Gentile, 2012; Cecchet & Thoburn, 2014; Platt et al., 2007; Shannon et al., 2008). In addition, since sex work is illegal, women in these occupations have little recourse to deal with physical and mental health problems.

Interestingly, we found that those who worked in both sex work and care/service work were more likely to have health concerns than women engaging exclusively in sex work. To our knowledge, there has been no research exploring the economic and health conditions of women who move between sex work and low-wage work. Further research is warranted to understand the social and economic context of women’s decision to move between these two types of jobs (Rosen & Venkatesh, 2008).

We also found that women who engaged in sex work had significantly higher self-esteem scores compared with women in care/service work only. This finding may reflect previous literature concerning sex work as a choice, in which women actively seek out alternative jobs to overcome their economic difficulties (Rosen & Venkatesh, 2008). The act of choosing to enter into better paying alternative work as an expression of agency can contribute to a sense of control. To be sure, this is not to argue that women are better off engaging in sex work than in low-wage care/service work. However, it may mean that scholars need to understand that women in difficult life situations retain agency in making decisions about their life.

Furthermore, although care/service work may protect women from exposure to the higher levels of physical and mental health problems encountered by women engaging in sex work, all the same, declining protection for workers and increasing contract, temporary, and other alternative work arrangements introduce insecurity and risk associated for low-wage workers (Kalleberg, 2011; Katz & Krueger, 2016). Low-wage jobs with few resources, invisibility, and vulnerability can no longer provide dignity and security for workers (Kennedy, 2010). Studies have documented that care workers are exposed to substantial job-related risks, including physical injuries and emotional burnout (Bureau of labor Statistics, 2016). Low-wage workers often face resource constraints, understaffing, and high turnover rates that lead to increased workplace injury and stress (Leigh, 2012; Underhill & Quinlan, 2011).

We acknowledge three important limitations. First, these data are self-reported, which may introduce bias, particularly with respect to questions on illicit activities involving substance use and sex work (Latkin, Ewards, Davey-Rothwell, & Tobin, 2017). Considering the setting, there may have been under-reporting of sex work, which would mean that our findings underestimate the negative effects of this work on health and wellbeing. Relatedly, our data did not allow us to take into account the length of time spent in a particular job. Due to this limitation, we were not able to examine the dose response relationship between job type and health outcomes.

Second, although we control for relevant sociodemographic variables in regression models, our analytic approach cannot fully determine how these differences might have shaped women’s work trajectories, at the same time, affecting health outcomes. Interestingly, we saw that women who worked in both sex and care/service work, compared with women working exclusively in sex work, had lower educational attainment and were more likely to have experienced ACEs.

Finally, our study characterizes experiences of a highly specific subgroup of women. We explored cross-sectional relationships between care/service work, sex work, incarceration, social support, and well-being focusing only on incarcerated women. However, our findings highlight several important issues that warrant further research to fully understand the relationship between paid care/service work, sex work, and poorer health outcomes for a larger population of women living in poverty.

Our findings add valuable insights to the current literature. The consequences of criminal justice involvement for men are well documented: poor employment opportunities, low-wages, and poor wage trajectories (Sykes & Geller, 2016; Western & Pettit, 2005; Western & Wildeman, 2009). However, these studies provide little information about the economic conditions and work experiences of incarcerated women. Research concerning the impact of low-wage care/service jobs needs to pay more attention to how female workers may move between formal and informal work, which, further marginalizes already economically disadvantaged women. Women in these low-wage jobs have fewer resources for dealing with health risks, and those who choose to engage in sex work are exposed to additional work-related risks, albeit in different ways than care/service work. Thus, there is a need for further evaluation of the impact of difficult life circumstances on occupational choices before incarceration. Clearly, incarceration diminishes future job prospects, but it could also be that socioeconomic factors that contribute to the risk of incarceration also determine type of work options available to individuals with a history of incarceration, even before their incarceration.

## Conclusion

While many traumatic events contribute to women’s entry into commercial sex work, economic challenges and other life circumstances need to be examined regarding how poor and unskilled incarcerated women navigate low-wage care work and sex work jobs (Stenning, 2005; Williams & Nadin, 2012). Many poor women involved in paid care/service work supplement their income through sex work. Boundaries between more legitimate, but low-wage, insecure care/service work jobs and the underground economy of illicit work may be more fluid than previously conceptualized. Notably, when women try to take control of their lives by leveraging benefits of these two types of work, the limited choices available to them tend to have negative effects on their mental and physical health and wellbeing.

## Data Availability

The data that support the findings of this study are available on reasonable request from the corresponding author SK. The data are not publicly available due to them containing information that could compromise research participant privacy and consent.

## Abbreviations

ACEs: Adverse Childhood Events
CCJ: Cook County Jail
CESD: Center for Epidemiologic Studies Depression Scale
FBI: Federal Bureau of Investigation
MOS: Medical Outcomes Study
PTSD: Post-traumatic stress disorder
UCR: Uniform Crime Report

## References

[CR1] Agustín LM, O'Neill M, Campbell R (2006). The conundrum of Women’s agency: Migrations and the sex industry. Sex work now.

[CR2] Alexander M (2010). The new Jim crow.

[CR3] American Psychological Association (2017). Center for Epidemiological Studies-Depression.

[CR4] Anklesaria A, Gentile J (2012). Psychotherapy with women who have worked in the “sex industry”. Innovations in Clinical Neuroscience.

[CR5] Bales K (1984). The dual labor market of the criminal economy. Sociological Theory.

[CR6] Bergseth K, Richardson Jens K, Bergerson-Vigesaa L, McDonal T (2011). Assessing the needs of women recently released from prison. Women & Criminal Justice.

[CR7] Bernstein E (2004). The transformation of sexual commerce and urban space in San Francisco.

[CR8] Binswanger I, Mueller S, Beaty B, Min S-J, Corsi K (2014). Gender and risk behaviors for HIV and sexually transmitted infections among recently released inmates: A prospective cohort study. AIDS Care.

[CR9] Brewer R (1999). Theorizing race, class and gender: The new scholarship of black feminist intellectuals and black Women's labor. Race, Gender & Class.

[CR10] Bureau of Justice Statistics (2018). Terms & Definitions: State and federal prisoners and prison facilities.

[CR11] Bureau of labor Statistics (2016). *Nonfatal occupational injuries and illnesses requiring days away from work, 2015*. Washington, DC: https://www.bls.gov/news.release/pdf/osh2.pdf. Accessed 13 June 2017.

[CR12] Bureau of Labor Statistics (2017). May 2016 National Occupational Employment and wage estimates United States.

[CR13] Burgard, S., & Lin, K. (2013). Bad jobs, bad health? How work and working conditions contribute to health disparities. *American Behavioral Scientist*, *57*(8). 10.1177/0002764213487347. 10.1177/0002764213487347 PMC3813007 24187340

[CR14] Cecchet S, Thoburn J (2014). The psychological experience of child and adolescent sex trafficking in the United States: Trauma and resilience in survivors. Psychological Trauma Theory Research Practice and Policy.

[CR15] Centers for Disease Control and Prevention. (2016). HIV Risk among persons who exchange sex for money or nonmonetary items. https://www.cdc.gov/hiv/group/sexworkers.html. Accessed 16 May 2020.

[CR16] Chen Y-H, McFarland W, Raymond HF (2011). Behavioral surveillance of heterosexual exchange-sex partnerships in San Francisco: Context, predictors and implications. AIDS and Behavior.

[CR17] Chicago Appleseed (2013). *Pretrial delay & length of stay in the Cook County jail*. Chicago: http://www.chicagoappleseed.org/wp-content/uploads/2012/06/CAFFJ-Pret-Trial-Delay-and-Length-of-Stay-Executive-Summary.pdf. Accessed 22 Sep 2020.

[CR18] Cobbina, J. (2009). *From prison to home: Women’s pathways in and out of crime*. Washington, DC: Accessed 22 Sep 2020.

[CR19] Cohan D, Kim A, Morrow R, Reardon J, Lynch M, Klausner JD (2005). Health indicators among low income women who report a history of sex work: The population based northern California young Women’s survey. Sexually Transmitted Infections.

[CR20] Cohan D, Kim A, Ruiz J, Morrow S, Reardon J, Lynch M (2005). Health indicators among low income women who report a history of sex work: The population based northern California young women’s study. Sexually Transmitted Infections.

[CR21] Cohan D, Lutnick A, Davidson P, Cloniger C, Herlyn A, Breyer J (2006). Sex worker health: San Francisco style. Sexually Transmitted Infections.

[CR22] Collins PH (2000). Black Feminist Thought.

[CR23] Cook County Department of Corrections (2019). *Sheriff's daily report*. Chicago: https://www.cookcountysheriff.org/wp-content/uploads/2019/12/CCSO_BIU_CommunicationsCCDOC_v1_2019_11_30.pdf. Accessed 22 Sep 2020.

[CR24] Cook County State's Attorney Office (2019). *Sentencing*. Chicago: https://datacatalog.cookcountyil.gov/Courts/Sentencing/tg8v-tm6u/data. Accessed 22 Sep 2020.

[CR25] Cox R (2012). The impact of mass incarceration on the lives of African American women. Review of Black Political Economy.

[CR26] Curtis MA (2011). The effect of incarceration on urban Fathers’ health. American Journal of Mens Health.

[CR27] Decker, S., Spohn, C., Ortiz, N., & Hedberg, E. (2014). *Criminal stigma, race, gender and employment: An expanded assessment of the consequences of imprisonment for employment*. Washington, DC: https://www.ncjrs.gov/pdffiles1/nij/grants/244756.pdf. Accessed 22 Sep 2020.

[CR28] Dwyer R (2013). The care economy? Gender, economic restructuring, and job polarization in the U.S. labor market. American Sociological Review.

[CR29] Edin K, Lein L (1997). Making ends meet: How single mothers survive welfare and low-wage work.

[CR30] Edlund L, Korn E (2002). An economic theory of prostitution. Journal of Political Economy.

[CR31] England P (2005). Emerging theories of care work. Annual Review of Sociology.

[CR32] England P, Budig M, Folbre, n. (2002). Wages of virtue: The relative pay of care work. Social Problems.

[CR33] Escobar G, Olson D (2012). A Profile of Women Released Into Cook County Communities from Jail and Prison.

[CR34] Fagan J, Freeman R (1999). Crime and work. Crime and Justice.

[CR35] Federal Bureau of Investigation (2000). *Uniform crime reports for the United States, 2000*. Washington, DC: http://www.fbi.gov/ucr/00cius.htm. Accessed 2 Nov 2006.

[CR36] Folbre N (2008). Reforming care. Politics and Society.

[CR37] Glenn EN (2010). Forced to care: Coercion and caregiving in America.

[CR38] Goffman A (2014). On the run.

[CR39] Gunter S (2017). Dynamics of urban informal labor supply in the United States. Social Science Quarterly.

[CR40] Harris R, Sharps P, Allen K, Anderson E, Soeken K, Rohatas A (2003). The interrelationship between violence, HIV/AIDS, and drug use in incarcerated women. Journal of the Association of Nurses in AIDS Care.

[CR41] Hays, R., Sherbourne, C., & Mazel, R. (1994). *User's manual for medical outcomes study (MOS) Core measures of health-related quality of life*. Santa Monica http://www.rand.org/pubs/monograph_reports/2008/MR162.pdf. Accessed 16 May 2020.

[CR42] Iceland J (2013). Poverty in America: A handbook.

[CR43] Jones A (2018). Correctional Control 2018: Incarceration and supervision by state.

[CR44] Kalleberg A (2011). Good jobs, bad jobs: The rise of polarized and precarious employment systems in the United States, 1970s to 2000s.

[CR45] Katz, L., & Krueger, A. (2016). *The rise and nature of alternative work arrangements in the United States, 1995–2015*. Cambridge: https://krueger.princeton.edu/sites/default/files/akrueger/files/katz_krueger_cws_-_march_29_20165.pdf. Accessed 15 May 2020.

[CR46] Kennedy E (2010). The invisible corner: Expanding workplace Rightsfor female day laborers. Berkeley Journal of Employment and Labor Law.

[CR47] Kilbourne B, England P, Farkas G, Beron K, Weir D (1994). Returns to skill, compensating differentials, and gender bias: Effects of occupational characteristics on the wages of White women and men. American Journal of Sociology.

[CR48] Kim S (2003). Incarcerated women in life context. Women's Studies International Forum.

[CR49] Kim S, Johnson TP, Goswami S, Puisis M (2011). Risk factors for homelessness and sex trade among incarcerated women: A structural equation model. Journal of International Women's Studies.

[CR50] Kinder, M. (2020). *Essential but undervalued: Millions of health care workers aren’t getting the pay or respect they deserve in the COVID-19 pandemic*. Washington, DC: https://www.brookings.edu/research/essential-but-undervalued-millions-of-health-care-workers-arent-getting-the-pay-or-respect-they-deserve-in-the-covid-19-pandemic/. Accessed 18 Sep 2020.

[CR51] Landsbergis P, Grzywacz J, LaMontagne A (2014). Work organization, job insecurity, and occupational health disparities. American Journal of Industrial Medicine.

[CR52] Latessa E (2012). Why work is important and how to improve the effectiveness of correctional reentry programs that target employment. Criminology & Public Policy.

[CR53] Latkin C, Ewards C, Davey-Rothwell M, Tobin K (2017). The relationship between social desirability bias and self-reports of health, substance use, and social network factors among urban substance users in Baltimore, Maryland. Addictive Behaviors.

[CR54] Leichliter J, Dittus P, Copen C, Aral S (2019). Trends in factors indicating increased risk for STI among key subpopulations in the United States, 2002–2015. Sexually Transmitted Infections.

[CR55] Leigh, J. P. (2012). *Numbers and costs of occupational injury and illness in low-wage occupations*. Davis: https://www.issuelab.org/resources/14420/14420.pdf. Accessed 10 May 2020.

[CR56] Levanon A, England P, Allison P (2005). Occupational feminization and pay: Assessing causal dynamics using 1950-2000 U.S. Census data. Social Forces.

[CR57] Levitt S, Venkatesh S (2008). An empirical analysis of street-level prostitution.

[CR58] Levitt S, Venkatesh SA (2000). An economic analysis of a drug-selling Gang's finances. Quarterly Journal of Economics.

[CR59] McCarthy B, Benoit C, Jansson M (2014). Sex work: A comparative study. Archives of Sexual Behavior.

[CR60] McClanahan S, McGlelland G, Abram K, Teplin L (1999). Pathways into prostitution among female jail detainees and their implications for mental health services. Psychiatric Services.

[CR61] Meyer MH (2000). Care work: Gender, labor, and the welfare state.

[CR62] Moller S, Rubin B (2008). The contours of stratification in service-oriented economies. Social Science Research.

[CR63] Morenoff J, Harding D (2014). Incarceration, prisoner reentry, and communities. Annual Review of Sociology.

[CR64] Murphy A, Venkatesh SA (2006). Vice careers: The changing contours of sex work in New York City. Qualitative Sociology.

[CR65] Nagin D, Waldfogel J (1995). The effects of criminality and conviction on the labor market status of young British offenders. International Review of Law and Economics.

[CR66] Nightingale, D., & Wandner, S. (2011). *Informal and nonstandard employment in the United States*. Washington, DC: https://www.urban.org/sites/default/files/publication/32791/412372-informal-and-nonstandard-employment-in-the-united-states.pdf. Accessed 22 Sep 2020.

[CR67] Noah T (2012). The great divergence.

[CR68] Nuytiens A, Christiaens J (2016). Female pathways to crime and prison: Challenging the (US) gendered pathways perspective. European Journal of Criminology.

[CR69] Okechukwu C, Souza K, Davis K, de Castro AB (2014). Discrimination, harassment, abuse, and bullying in the workplace: Contribution of workplace injustice to occupational health disparities. American Journal of Industrial Medicine.

[CR70] Olson, D., & Huddle, K. (2013). *An examination of admissions, discharges & the population of the Cook County jail, 2012*. Chicago: https://ecommons.luc.edu/cgi/viewcontent.cgi?article=1015&context=social_justice. Accessed 27 Jan 2020.

[CR71] Olson, D., & Taheri, S. (2012). *Population dynamics and the characteristics of inmates in the Cook County jail*. Chicago: http://ecommons.luc.edu/cgi/viewcontent.cgi?article=1000&context=criminaljustice_facpubs. Accessed 27 Jan 2020.

[CR72] Park JN, Gaydos C, White RH, Decker M, Footer K, Galai N (2019). Incidence and predictors of chlamydia, gonorrhea and Trichomonas among a prospective cohort of Cisgender female sex Workers in Baltimore, Maryland. Sexually Transmitted Diseases.

[CR73] Paz-Bailey G, Noble M, Salo K, Tregear S (2016). Prevalence of HIV among US female sex workers: Systematic review and meta-analysis. AIDS and Behavior.

[CR74] Platt L, Rhodes T, Judd A, Koshkina E, Maskimova S, Latishevskaya N (2007). Effects of Sex Work on the Prevalence of Syphilis Among Injection Drug Users in 3 Russian Cities. American Journal of Public Health.

[CR75] Puri, N., Shannon, K., Nguyen, P., & Goldenberg, S. (2017). Burden and correlates of mental health diagnoses among sex workers in an urban setting. *BMC Women's Health*, *17*(133). 10.1186/s12905-12017-10491. 10.1186/s12905-017-0491-y PMC5735638 29258607

[CR76] Rabuy, B., & Kopf, D. (2015). *Prisons of poverty: Uncovering the pre-incarceration incomes of the imprisoned*. Northampton: https://www.prisonpolicy.org/reports/income.html. Accessed 22 Sep 2020.

[CR77] Raphael J, Shapiro D (2002). Sisters speak out.

[CR78] Riley E, Gandhi M, Hare B, Cohen J, Hwang S (2007). Poverty, unstable housing, and HIV infection among women living in the United States. Current HIV/AIDS Reports.

[CR79] Romero M, Pérez N (2016). Conceptualizing the Foundation of Inequalities in care work. American Behavioral Scientist.

[CR80] Rosaldo M, Tilly C, Evans P (2012). A conceptual framework on informal work and informal worker organizing.

[CR81] Rosen E, Venkatesh SA (2008). A “perversion” of choice sex work offers just enough in Chicago's urban ghetto. Journal of Contemporary Ethnography.

[CR82] Salisbury E, Van Voorhis P (2009). Gendered pathways: A quantitative investigation of women Probationers’ paths to incarceration. Criminal Justice and Behavior.

[CR83] Sampson R, Laub J (1993). Structural variations in juvenile court processing: Inequality, the underclass, and social control. Law and Society Review.

[CR84] Sentencing Project. (2012, December 2012). Incarcerated women. http://www.sentencingproject.org/doc/publications/cc_Incarcerated_Women_Factsheet_Dec2012final.pdf. Accessed 16 Jan 2014.

[CR85] Shannon K, Kerr T, Allinott S, Chettiar J, Shoveller J, Tyndall M (2008). Social and structural violence and power relations in mitigating HIV risk of drug-using women in survival sex work. Social Science & Medicine.

[CR86] Sherbourne C, Stewart A (1991). The MOS social support survey. Social Science & Medicine.

[CR87] Smith, V., & Halpin, B. (2011). Low-wage Work Uncertainty often Traps Low-wage Workers https://poverty.ucdavis.edu/sites/main/files/file-attachments/smith_cpr_policy_brief_employability.pdf. Accessed 19 Sept 2020.

[CR88] Solomon, A., Deadel Johnson, K., Travis, J., & McBride, E. (2004). *From Prison to Work: The Employment Dimensions of Prisoner Reentry*. Washington, DC: https://www.urban.org/sites/default/files/publication/58126/411097-From-Prison-to-Work.PDF. Accessed 19 Sept 2020.

[CR89] Steege A, Baron S, Marsh S, Chaumont Menendez C, Myers J (2014). Examining occupational health and safety disparities using national data: A cause for continuing concern. American Journal of Industrial Medicine.

[CR90] Steinberg R (1990). Social construction of skill: Gender, power, and comparable worth. Work and Occupations.

[CR91] Stenning A (2005). Re-placing work: Economic transformation and the shape of a community in post-socialist Poland. Work, Employment and Society.

[CR92] Sullivan R (1973). The economics of crime: An introduction to the literature. Crime and Delinquency.

[CR93] Sykes B, Geller A (2016). Mass Incarceration and the Underground Economy in America.

[CR94] The Sentencing Project (2016). *Trends in US corrections*. Washington, DC: http://sentencingproject.org/wp-content/uploads/2016/01/Trends-in-US-Corrections.pdf. Accessed 25 Sep 2020.

[CR95] The Urban Justice Center (2005). Behind Closed Doors: An analysis of indoor sex work in New York City.

[CR96] Uggen C, Manza J (2002). Democratic contraction? Political consequences of felon disenfranchisement in the United States. American Sociological Review.

[CR97] Underhill E, Quinlan M (2011). How precarious employment affects health and safety at work: The case of temporary agency workers. Industrial Relations.

[CR98] Venkatesh S (2006). Off the books.

[CR99] Weitzer R (2009). Sociology of sex work. Annual Review of Sociology.

[CR100] Weitzer R (2010). The movement to criminalize sex work in the United States. Journal of Law and Society.

[CR101] Western B (2002). The impact of incarceration on wage mobility and inequality. American Sociological Review.

[CR102] Western B (2007). Mass imprisonment and economic inequality. Social Research.

[CR103] Western B, Beckett K (1999). How unregulated is the U.S. labor market? The penal system as a labor market institution. American Journal of Sociology.

[CR104] Western B, Pettit B (2005). Black-White wage inequality, employment rates, and incarceration. American Journal of Sociology.

[CR105] Western B, Wildeman C (2009). The black family and mass incarceration. Annals of the American Academy of Social and Political Science.

[CR106] Williams C, Nadin S (2012). Work beyond employment: Representations of informal economic activities. Work, Employment and Society.

[CR107] Wright E, Van Voorhis P, Salisbury E, Bauman A (2012). Gender-responsive lessons learned and policy implications for women in prison: A review. Criminal Justice and Behavior.

